# Palatal myoclonus secondary to neurosarcoidosis

**DOI:** 10.1002/ccr3.2619

**Published:** 2020-02-03

**Authors:** Devanshi Dharaiya, Anza B. Memon

**Affiliations:** ^1^ Department of Neurology Henry Ford Hospital Detroit MI USA; ^2^ School of Medicine Wayne State University Detroit MI USA

**Keywords:** demyelination, Guillain and Mollaret triangle, neurosarcoidosis, palatal myoclonus

## Abstract

Palatal myoclonus can be primary or secondary. In primary palatal myoclonus, no obvious structural brain lesions can be found within the triangle of Guillain and Mollaret. Common causes of secondary myoclonus include stroke, demyelination, infections, trauma, and neurodegeneration.

## Graphical abstract

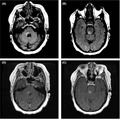

A 49‐year‐old woman with remote history of pulmonary sarcoidosis presented with gait and speech problems. Neurological examination showed dysarthria, left upper and lower extremity dysmetria, truncal ataxia, and perioral myokymia. Magnetic resonance imaging of the brain revealed a hyperintense lesion within the right cerebellar peduncle extending up into the pons on fluid‐attenuated inversion recovery sequence demonstrating enhancement, affecting the Guillain and Mollaret triangle (Figure 1).

**Figure 1:**
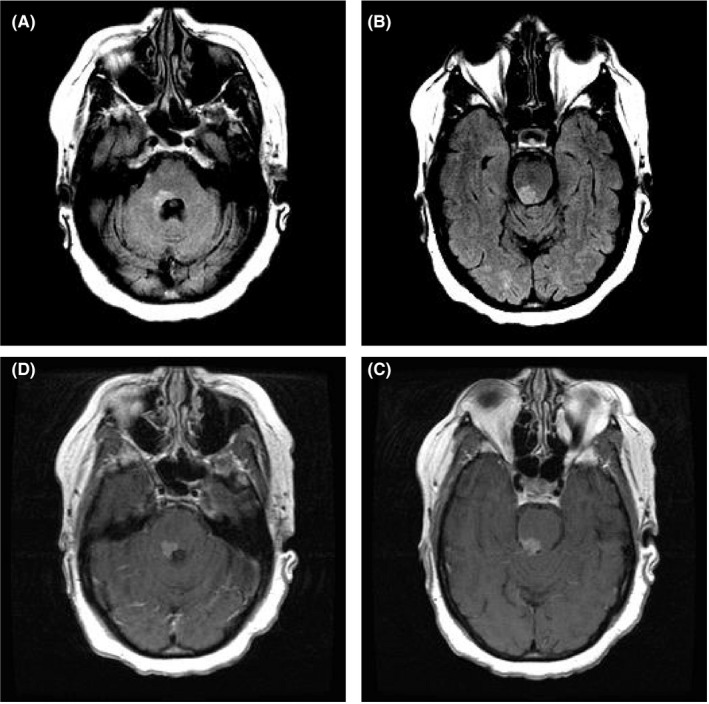
T2 hyperintensity seen within the right cerebellar peduncle (A) extending up into the pons (B) demonstrating enhancement (C and D)

Three months later, the patient developed palatal myoclonus (see Video S1) which was noted on examination. The patient was unaware of the involuntary palatal movement and denied any clicking sound in her ears.

Palatal myoclonus can be primary or secondary. In primary palatal myoclonus, no obvious structural brain lesions can be found within the triangle of Guillain and Mollaret.^1^ Common causes of secondary myoclonus include stroke, demyelination, infections, trauma, and neurodegeneration.^2^

## CONFLICT OF INTEREST

None declared.

## AUTHOR CONTRIBUTIONS

ABM: involved in conception and design, acquisition of data, analysis and interpretation of data, drafting the article, revising it critically for important intellectual content, and final approval of the version to be published. DD: contributed to conception, design, data collection, and interpretation of data.
